# Within-Population Genome Size Variation is Mediated by Multiple Genomic Elements That Segregate Independently during Meiosis

**DOI:** 10.1093/gbe/evz253

**Published:** 2019-11-19

**Authors:** Claus-Peter Stelzer, Maria Pichler, Peter Stadler, Anita Hatheuer, Simone Riss

**Affiliations:** Research Department for Limnology, University of Innsbruck, Mondsee, Austria

**Keywords:** genome size, genetic variation, rotifer, C-value paradox, junk DNA, B-chromosomes

## Abstract

Within-species variation in genome size has been documented in many animals and plants. Despite its importance for understanding eukaryotic genome diversity, there is only sparse knowledge about how individual-level processes mediate genome size variation in populations. Here, we study a natural population of the rotifer *Brachionus asplanchnoidis* whose members differ up to 1.9-fold in diploid genome size, but were still able to interbreed and produce viable offspring. We show that genome size is highly heritable and can be artificially selected up or down, but not below a certain basal diploid genome size for this species. Analyses of segregation patterns in haploid males reveal that large genomic elements (several megabases in size) provide the substrate of genome size variation. These elements, and their segregation patterns, explain the generation of new genome size variants, the short-term evolutionary potential of genome size change in populations, and some seemingly paradoxical patterns, like an increase in genome size variation among highly inbred lines. Our study suggests that a conceptual model involving only two variables, 1) a basal genome size of the population, and 2) a vector containing information on additional elements that may increase genome size in this population (size, number, and meiotic segregation behavior), can effectively address most scenarios of short-term evolutionary change of genome size in a population.

## Introduction

Despite an exponential increase of genomic information during the last two decades, there is still no consensus about the ultimate causes of genome size variation in eukaryotes (Cavalier-Smith 2005; Gregory 2005; Lynch 2007). At the core of this controversy is the puzzling genome size variation across eukaryotic taxa, spanning approximately five orders of magnitude. Genome sequencing has revealed that this variation is primarily caused by gene-, chromosome-, or genome duplications, by variation in the length of introns, number of transposons, and the amount of simple repetitive DNA (Kidwell 2002; Bennetzen et al. 2005; Bennetzen and Wang 2014; Elliott and Gregory 2015). Since most of these sequences make no substantial contribution to the phenotype, at least not through their information content, genome size is only a poor predictor of organismal complexity in eukaryotes (Gregory 2005). On the other hand, ubiquitous correlations of genome size and cell size, or other phenotypic traits such as metabolic- or developmental rates, and body size (Gregory 2001, 2002; Beaulieu et al. 2008; Dufresne and Jeffery 2011; Realini et al. 2016), suggest the sheer amount of DNA in a genome can affect the phenotype (Bennett 1972). Collectively, this might explain why current theories on genome size variation in eukaryotes differ strongly in their emphasis on selection, mutation, and drift (Bennett 1972; Petrov 2002; Cavalier-Smith 2005; Lynch 2007; Hessen et al. 2010; Hessen 2015).

Theories invoking selection for increased genome size have been criticized for assuming causal links behind the correlations between genome size and phenotypic traits (Lynch 2007). Indeed, such correlations often involve species that have been separated for long evolutionary timespans and thus differ in many other aspects than genome size. According to the mutational hazard hypothesis, noncoding DNA is never beneficial, but it may accumulate as a consequence of genetic drift (Lynch and Conery 2003). Thus, this hypothesis offers an alternative, neutral explanation to the observed genome size—phenotype correlations by stating that the accumulation of noncoding DNA in organisms with large body size might be due to their smaller effective population sizes (Lynch and Conery 2003; Lynch et al. 2011). Testing whether large genome size can sometimes be beneficial, or whether it is at least conditionally deleterious, ideally requires a model system that exhibits substantial genome size differences across a relatively homogeneous genomic background. This requirement appears to be best fulfilled in species with intraspecific genome size variation, where individuals share their genomic background and evolutionary history.

Cases of intraspecific genome size variation are well-documented in plants (Smarda and Bures 2010), with cultivated maize and its close relatives being probably one of the best-studied examples (Chia et al. 2012; Díez et al. 2013). In animals, intraspecific genome size variation has been found in snapping shrimp (Jeffery et al. 2016) and in grasshoppers (Ruiz-Ruano et al. 2011). Interestingly, two intensively studied model species with comparably small genomes, *Arabidopsis thaliana* and *Drosophila melanogaster*, have also turned out to exhibit substantial levels of intraspecific genome size variation (Long et al. 2013; Huang et al. 2014), suggesting that this phenomenon might be more widespread than previously assumed. Intraspecific genome size variation is sometimes associated with variation in chromosome numbers, for instance due to supernumerary (B-)chromosomes (Smarda and Bures 2010), but there are also documented cases where genome size variation is not reflected in the karyotype (Šmarda et al. 2008; Jeffery et al. 2016).

Despite its importance, surprisingly little is known about the basic mechanisms and inheritance of intraspecific genome size variation, and the links to population-level phenomena, that is: What characterizes those parts of a genome that account for the differences in genome size between individuals? How is genome size inherited by offspring? How (fast) can the trait “genome size” change through generations, for example, if it is directionally selected? Even in the best-studied systems, researchers typically rely on assumptions and draw analogies to models of quantitative genetic variation. For example, the trait “genome size” is often considered a quantitative trait (QT) influenced by a large number “loci,” with “alleles” that increase or decrease genome size (Šmarda et al. 2010; Bilinski et al. 2018). We are not aware of any empirical study that has yet assessed the appropriateness of such a model.

Here, we study using flow cytometry the basic mechanisms of genome size variation in a population of the monogonont rotifer *Brachionus asplanchnoidis*. This species is characterized by an almost two times larger genome size relative to its sister species, *Brachionusplicatilis* and *Brachionusmanjavacas* (Stelzer et al. 2011), by a high 44% genomic content of repetitive elements (Blommaert et al. 2019), and by intraspecific genome size variation (Stelzer et al. 2011; Mills et al. 2016). Monogonont rotifers of the genus *Brachionus* are cyclical parthenogens, that is, they alternate between ameiotic parthenogenesis and sexual reproduction. A *quorum-sensing* chemical released at high population densities (Kubanek and Snell 2008) triggers the production of sexual females, whose oocytes undergo meiosis and develop into haploid males (if not fertilized) or diploid diapausing eggs (if fertilized). Self-fertilization is possible, if males mate with sexual females of the same clone, even though this should be a rare event in genetically diverse populations. Using crossbreeding experiments, selfed lines, and artificial selection, we disentangle the basic mechanism by which variation for genome size is mediated in this population, and how it is inherited by offspring. We capitalize on several advantages of our model system, such as short generation times, sexual and asexual reproduction, and a haploid–diploid lifecycle, which allows us to probe into meiotic patterns associated with intraspecific genome size variation. We could identify the size and number of individual elements that contribute to increases in genome size, and thus account for the gradual differences among individuals in the population. Our findings suggest that intraspecific genome size variation can be conceptualized in terms of a basal genome size (representing the smallest genome size attainable in a population), and additional “elements” found in individuals with larger genomes (each characterized by size, number, and meiotic segregation behavior). This difference in perspective, compared with a conventional QT model, has significant implications on the genome size distribution of populations, and the short-term evolutionary potential of the trait “genome size.”

## Materials and Methods

Resting eggs of rotifers were collected in the field from Obere Halbjochlacke (OHJ, N 47°47′11″, E 16°50′31″) and from Runde Lacke (N 47°47′08″, E 16°47′34″), two small alkaline playa lakes in Burgenland (Austria) in 2011. Resting eggs from Lake Nakuru (Kenya) and the two Mongolian clones were obtained from colleagues and have been previously described in detail (Stelzer et al. 2011; Riss et al. 2017). All rotifers were cultured as clones, consisting of the asexual descendants of the female that initially hatched from a resting egg (for details, see supplementary methods, Supplementary Material online).

Genome size measurements were performed with flow cytometry using a detergent-trypsin method and propidium iodide (PI) staining according to (Stelzer et al. 2011) with minor modifications (for details, see supplementary methods, Supplementary Material online). As an internal standard of known genome size, we used the fruit fly, *Drosophila melanogaster* (strain ISO-1, diploid nuclear DNA content: 0.35 pg; Gregory 2019).

For sexual crosses between two rotifer clones, we used freshly hatched virgin females and males, which were harvested as eggs from dense rotifer cultures that had initiated sexual reproduction (for details, see supplementary methods, Supplementary Material online). To analyze inheritance of genome size within a population, we crossed two clones with divergent diploid genome size (414 vs. 524 Mb; called ohj22 and ohj7, respectively) and analyzed 27 of their sexual offspring. Two selfed lines were established from the same two clones by growing mass cultures until they produced resting eggs (which were the product of self-fertilization, i.e., males fertilizing females of the same clone). Selfed lines were propagated for three sexual generations by randomly selecting one offspring clone each generation. Finally, we randomly selected one offspring clone from the “large” and “small” line and cross-mated them to produce an interline cross.

For the artificial selection experiments, we applied truncation selection to the natural OHJ population by crossing eight clones representing the 10% largest genome sizes among each other (excluding the outlier clone at 792 Mb), and by crossing eight clones representing the 10% smallest genomes, respectively. In the first generation, we used 14 combinations of parental clones for each selection treatment and analyzed 1–9 of their offspring. In total, the F1-generation encompassed 31 and 26 offspring clones for the large and small selection line, respectively. We repeated this selection procedure in the F1-generation to produce a F2-generation. In the small selection line, we used five clone combinations to produce 15 F2-offspring clones. In the large selection line, sexual propensity of the F1-clones was extremely low, limiting us to just one clone combination and 14 of their offspring in F2. The exact genealogy of clones in the F1- and F2-generation can be inferred from their names listed in supplementary table 1, Supplementary Material online. Narrow sense heritabilities *h*^2^ were estimated using to the “Breeder’s equation” Δ*Z* = *h*^2^ · *S*, where *S* is the selection differential, and Δ*Z* is the response to selection. Additionally, we calculated *h*^2^ from the slope of the best-fit line for a plot of midoffspring versus midparent genome size of all our available data, which included all crosses and the self-fertilized clones.

To determine the genome size of males relative to (diploid) females, we grew clones from low to high population densities until they started to produce males. We coprepared males and females from the same clone and subjected them to the same protocol as above, except that we did not use a *Drosophila* internal standard. To better visualize male peaks, we used an excess of males, from 200 males + 100 females to 300 males + 60 females, depending on genome size of the clone. In the flow-cytometry analysis, we quantified the following variables: number of male peaks (up to 6), position of each individual male peak (as the median of the YL-2A value), position of the female peak, SD (YL-2A value) of all combined male peaks, and SD of the female peak. In contrast to the previous genome size measurements, we applied a slightly stricter precision cut-off at 3.5% coefficient of variance (CV) of the diploid female peak, and discarded all samples with higher CVs.

We performed two types of analyses depending on the quality our male peak data. First, in clones that showed multiple discrete male peaks, we counted and sized these peaks (relative to the female peak). We also determined the area under each peak, as a measure for the frequency of each male genome size class. Second, for these and for all remaining clones, we calculated relative coefficient of variation (RCV) according to:

$$\mathrm{RCV}={\mathrm{CV}}_{\mathrm{allMP}}/{\mathrm{CV}}_{\mathrm{FP},}$$

where *CV*_allMP_ is the CV across all male peaks and *CV*_FP_ is the CV of the female peak. Thus, RCV is a measure for male genome size variation within a sample, corrected for its measurement error (indicated by the CV of the female peak). To obtain a point of reference for RCV values in species without intraspecific genome size variation, we conducted the same analyses in four sister species (*Brachionusrotundiformis*, *B.plicatilis*, *B.manjavacas*, B. “Nevada”).

## Results

### Within-Population Genome Size Variation in *Brachionus asplanchnoidis*

**Fig. 1.**
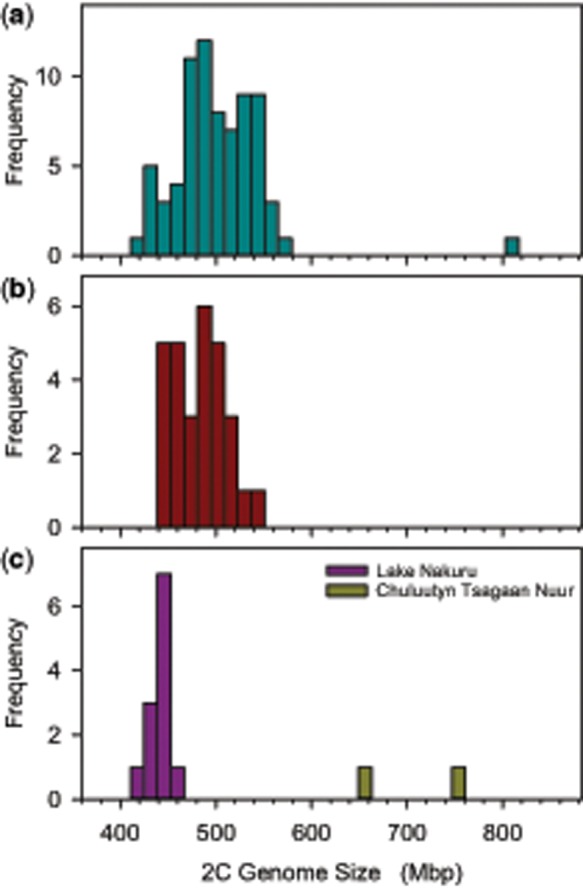
—Genome size variation in four natural *Brachionus asplanchnoidis* populations. (*a*) Obere Halbjochlacke (Austria). (*b*) Runde Lacke (Austria). (*c*) Lake Nakuru (Kenya) and Chuluutyn Tsagaan Nuur (Mongolia). Genome size was highly variable in the two Austrian populations (genome sizes ranging from 414 to 792 Mb), whereas clones isolated from Lake Nakuru sediments differed little from each other (426 ± 5.1 Mb, mean and SD). The two clones from the Mongolian site had distinct and relatively large genome sizes of 652 and 732 Mb (data from Riss et al. 2017).

To quantify genome size variation within populations, we examined 118 *B. asplanchnoidis* clones sampled from four geographic populations (fig. 1 and supplementary table 1, Supplementary Material online). Of these, two Austrian populations from “Obere Halbjochlacke” (OHJ, 74 clones) and “Runde Lacke” (RL, 29 clones) show highly significant intrapopulation variation (OHJ: ANOVA *F*_52,__194_ = 112, *P *<* *0.001; RL: *F*_28,__84_ = 23.14, *P *<* *0.001). The OHJ-population spans a genome size range of 1.33-fold, from 414 to 552 Mb (fig. 1*a*), with one outlier at 792 Mb (i.e., 1.91-fold). The RL-population is also variable (fig. 1*b*), spanning a range of 1.24-fold across all sampled clones. In contrast, the Lake Nakuru population (11 clones sampled) was not significantly variable (*F*_10,__21_ = 8.64, *P *=* *0.078), and most genomes were close to 420 Mb (fig. 1*c*). Previously, we reported two conspecific clones isolated from a Mongolian lake, which have relatively large genome sizes of 652 and 732 Mb (fig. 1*c*, data from Riss et al. 2017). Genome size is mitotically stable, since the genome sizes of clones, as well as the differences among clones, were highly reproducible over a period of >5 years (i.e., ∼600 asexual generations, supplementary fig. 1, Supplementary Material online).

### Inheritance of within-Population Genome Size Variation

**Fig. 2.**
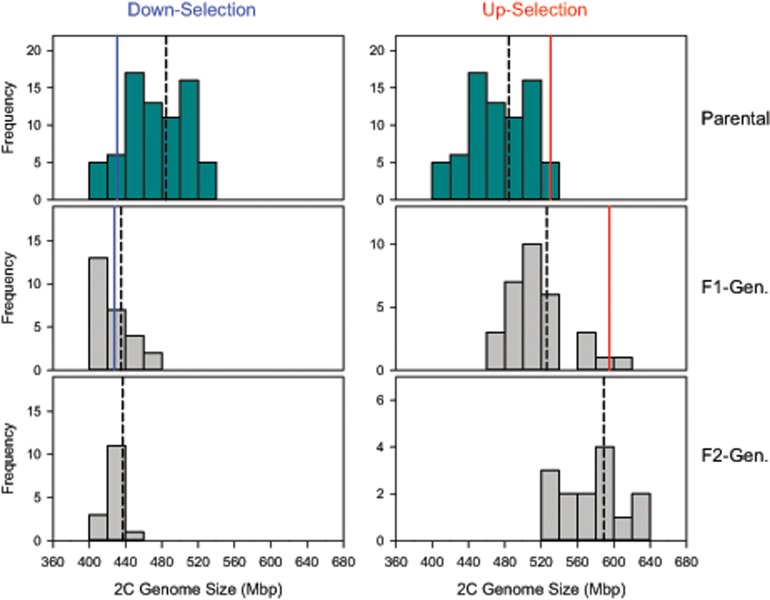
—Artificial selection for increased or reduced genome size. The parental population consisted of clones isolated from the natural population from Obere Halbjochlacke (cf., fig. 1*a*) and was the same for both treatments. Dashed black lines indicate population mean genome sizes for each generation. Solid lines indicate the mean genome size of the selected top 10% (in red) or bottom 10% (in blue) for the parental and F1-generation.

Clones with divergent genome size can mate with each other and produce viable and fertile offspring, which are intermediate in genome size between their parents but show some variation (supplementary fig. 2, Supplementary Material online, see also Riss et al. 2017). Genome size responds to artificial selection with extremely high heritability. We applied truncation selection to the OHJ population by crossing clones with the 10% largest genome sizes among each other (excluding the outlier clone at 792 Mb), and by crossing clones with the 10% smallest genomes, respectively. In the up-selection treatment, we obtained genome sizes exceeding the range of the parental OHJ population (fig. 2). We could select genome sizes of up to 640 Mb, with a narrow-sense heritability *h*^2^ of 0.905 in the first generation, and 0.912 in the second generation. Likewise, heritability was high in the first generation of the down-selection treatment (*h*^2^ = 0.924). However, in contrast to selection for large genome size, it did not extend the range of the original population. In fact, we could not select genome sizes <414 Mb. Additionally, *h*^2^ of the second generation of the down-selection treatment collapsed to zero. A parent–offspring regression including all our crosses yields an overall estimate for *h*^2^ of 0.96 (supplementary fig. 3, Supplementary Material online).

Combining all available genome size estimates of *B. asplanchnoidis* reveals a positively skewed distribution, with a high number of observations at 410–430 Mb and an elongated tail of large genome sizes (supplementary fig. 4, Supplementary Material online). The striking absence of genome sizes smaller than 410 Mb, despite high sampling effort and intentional selection for small genome size, suggests that this genome size might be a true biological limit. By contrast, there was no constraint in terms of increases in genome size, as suggested by the more or less continuous rise of genome sizes to 792 Mb. Interestingly, this clone with the largest genome size was not artificially selected, but hatched from a resting egg of the natural population (c.f., fig. 1*a*).

**Fig. 3.**
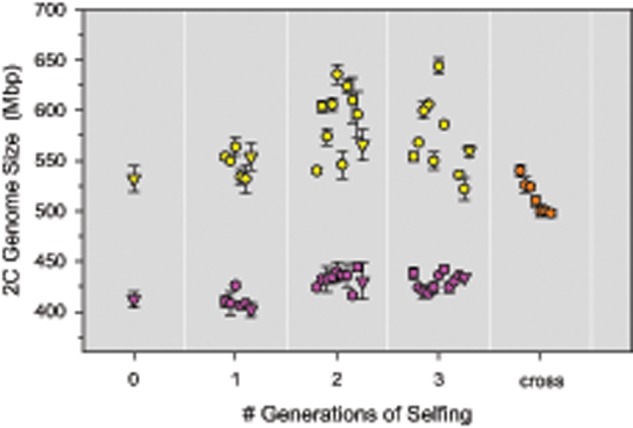
—Genome size variation in two selfed lines and their interline cross. Two selfed lines were established from two natural rotifer clones, one with large genome size (524 Mb, yellow) and one with small genome size (414 Mb, purple), and were propagated for three sexual generations. Clones used for breeding are indicated by triangles, their siblings are displayed as circles. After three generations of selfing, the two lines were crossed with each other to produce hybrid offspring (orange).

To get additional insights into the inheritance of genome size, we analyzed two selfed lines that were derived from a clone with large and small genome size, respectively. Theoretically, one would expect trait variation to decrease upon selfing as this causes 50% reduction of heterozygosity each generation. In contrast, we found that genome size variation remained high in a selfed line that descended from a large-sized clone (524 Mb). Genome sizes of selfed offspring ranged between values of 522 and 644 Mb, representing a 23% increase (fig. 3). Among-clone variation in the large selfed line was statistically significant, even after three generations of selfing (ANOVA *F*_9,__24_ = 53.61, *P *<* *0.001). Variation in the large line was also significantly higher than in the line descending from the small genome (*P *<* *0.001; R package *cvequality*, version 0.2.0; Marwick and Krishnamoorthy 2019). After three generations of selfing, we performed a sexual cross between both lines. As was the case in the cross between two natural clones, the cross of the selfed lines yielded offspring that were variable and intermediate between their parents (fig. 3).

### Evidence for Independently Segregating Genomic Elements

**Fig. 4.**
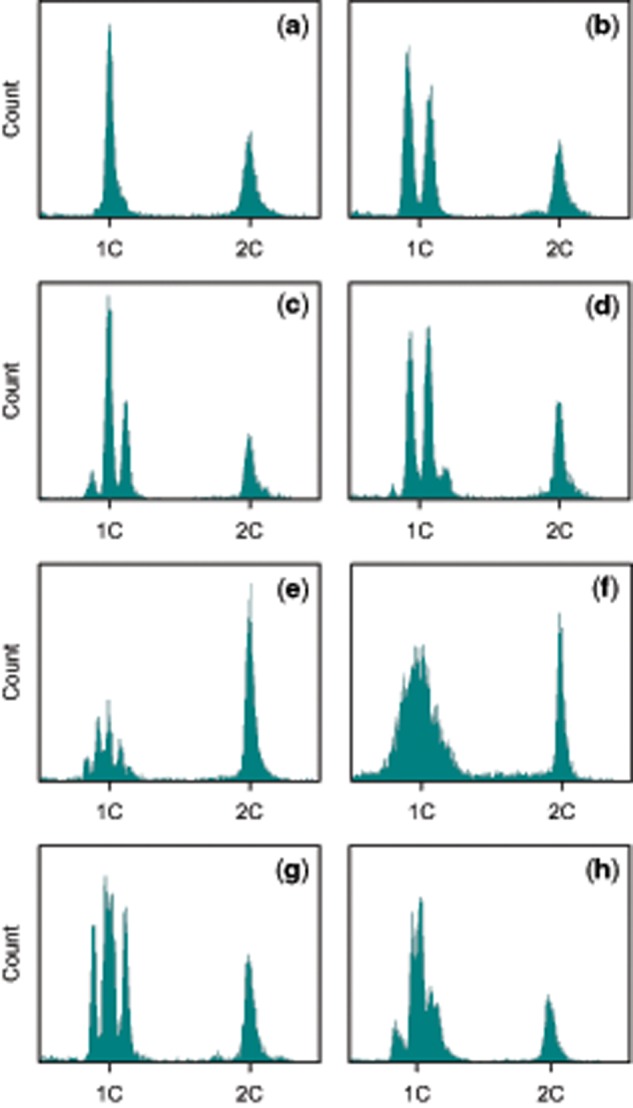
—Representative examples of genome size variation in haploid males. Each histogram corresponds to a different clone. *X* axis corresponds to the YL2-A fluorescence on a linear scale. Only the section surrounding the 1 C and 2 C values is shown. The 1 C value was set at 0.5× the median fluorescence of the 2 C (female) peak. (*a*) One single “male peak” (MP), indicates that male genome size is not variable. Note that the MP is located at exactly one half (=1 C) of the diploid female genome size (2 C). (*b*) Two MPs located at 0.46 and 0.54 of female genome size, indicating that there are two discrete classes of male genome size. (*c*) Three MPs, located at 0.44, 0.5, and 0.56. (*d*) Four MPs, located at 0.41, 0.47, 0.53, and 0.59. (*e*) Five MPs, located at 0.42, 0.46, 0.5, 0.54, and 0.58 (the MP on the far right is very faint). (*f*) No discrete MPs, but extremely broad distribution of male genome sizes around the 1 C value. However, the 2 C peak of this sample is very narrow (∼2% CV), indicating that the broad MP is due to real variation in genome size rather than a lack of precision. (*g*) Four MPs. Note that in contrast to (*d*), the MPs are approximately in the same height (i.e., relative frequency), and that there is unequal spacing between the MPs (the middle MPs are close together). (*h*) Six MPs with unequal spacing, showing some resemblance to the case of three MPs, but each MP appears to be split up into two subpeaks.

To gain mechanistic insights into segregation of genome size variation during meiosis, we analyzed haploid rotifer males. In a diploid organism with equally sized chromosome pairs and no extrachromosomal elements, all meiotic products should contain exactly half the DNA of a diploid cell. Consistently, haploid males of the rotifer *Brachionuscalyciflorus* have half the genome size of females (Stelzer et al. 2010; see Appendix S2 in this publication). We also find this pattern in some of our *B. asplanchnoidis* clones (e.g., fig. 4*a*). However, in many others, we obtained striking variation in male genome size, which manifested in multiple discrete “male peaks” (fig. 4*b*–*h* and supplementary table 2, Supplementary Material online). In the simplest case, we observed two male peaks spaced symmetrically around the expected 1 C-value (fig. 4*b*). In general, the male peak pattern of a clone could be characterized by 1) the number of peaks, 2) an odd/even number of peaks, indicating the presence/absence of a central male peak at exactly 1 C, and 3) the relative abundance of certain male genome size classes, as inferred from the area under each male peak. In most cases, the central male genome sizes (the ones close to 1 C) were more abundant than the ones in the periphery (supplementary fig. 5 and table 2, Supplementary Material online). Overall, male peak patterns were clone-specific and highly repeatable (supplementary fig. 6, Supplementary Material online).

In some clones, we could not resolve individual male peaks, but instead obtained an extremely broad distribution of genome sizes around the 1 C value (e.g., fig. 4*f*). This pattern likely reflects real genome size variation among males of a clone, rather than a lack of measurement precision, since the coefficient of variation of male genome sizes in this example was 12%, while variation of the female peak ∼2%. Interestingly, this clone with the extremely broad male peak was the same as the “outlier” with the largest genome size of 792 Mb (fig. 1*a*). Although this clone was the extreme case, we obtained broad distributions of male genome sizes in other clones as well (data not shown). Finally, in some clones, we observed characteristically unequal distances between male peaks (fig. 4*g* and *h*), such that some male peaks were closer together than others.

All the subsequent results and analyses build on the following ad hoc hypothesis, which is based on our observations so far: We hypothesized that large genomic elements, several Mb in size, are segregating independently from each other, and thus give rise to discrete classes of male genome size. By independent, we mean that each element has an approximately equal chance of segregating into any of the four gametes during gametogenesis. Note that this is different from normal chromosome segregation, where homologous chromosomes will always end up in opposite gametes. The presence of one independently segregating element can explain the simplest pattern of two male peaks (fig. 4*b*), being present in the large male genome size but absent in the small one. Similarly, two equally sized elements can explain a pattern of three male peaks (fig. 4*c*), which correspond to genome size classes with zero, one, or two elements. Likewise, patterns with higher numbers can be explained by *n*−1 elements. Assuming that independently segregating elements have identical size, we can predict the relative ratios of the male genome size classes to 1:1 (one element), 1:2:1 (two elements), or 1:3:3:1 (three elements). Indeed, some of our clones closely follow these predicted frequencies, whereas others showed some deviations (supplementary fig. 5, Supplementary Material online). For example, in clones with two male peaks, the smaller male genome size was often at higher frequency than the expected value of 0.5 (supplementary fig. 5, Supplementary Material online), suggesting that males without an element were more frequent among the hatched males. Likewise, in clones with three and four male peaks, the central male genome sizes were sometimes at a higher frequency than expected. One clone showing four male peaks with almost identical heights (supplementary fig. 5*c*, Supplementary Material online) presents a particularly interesting deviation. In this same clone, the two central peaks were closer together (cf. fig. 4*g*). The most parsimonious explanation seems to be that this clone carries two differentially sized elements, a small and a large one, and that the four male peaks correspond to: 1) zero elements, 2) the small element, 3) the large element, and 4) both elements. Likewise, two large and one small element can explain the “three double-peaks” pattern in the clone depicted in figure 4*h*.

**Fig. 5.**
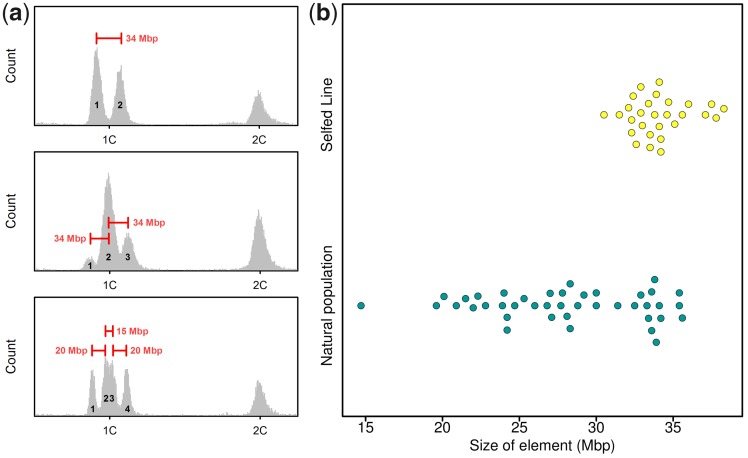
—Sizing of genomic elements responsible for male genome size variation. (*a*) Three examples of size inference, based on the distance between two male peaks (MPs) and knowledge of diploid genome size. Top: Example of a clone with two MPs, which can be explained by segregation of one ∼34 Mb element. Males corresponding to peak 2 contain the 34 Mb element, while males corresponding to peak 1 are lacking it. Middle: Example of a clone with three MPs, which can be explained by two elements of 34 Mb size: Males corresponding to peak 1 are free of elements, males of peak 2 contain one element, and males of peak 3 contain both elements. Bottom: A more complicated case with two, apparently differently sized elements (20 and 35 Mb): Peak 1 corresponds to males without any element, peak 2 to males with the 20 Mb element, peak 3 to males with the 35 Mb element, and peak 4 to males with both elements. (*b*) Beeswarm plot showing the estimated sizes of elements in the natural population (including their outbred offspring) and in the selfed line (yellow).

With this in mind, we estimated the size and number of independently segregating elements, based on the distance between male peaks in a clone and its 2 C genome size (fig. 5*a*). We find that the natural OHJ-population harbors a large diversity of elements (fig. 5*b*). Many clones contain elements of ∼34 Mb size, but smaller elements down to 15 Mb were also present in this population. In contrast, the clones of our “large” selfed line apparently contained only elements in the 34 Mb size range (fig. 5*b*), and they exhibited significantly less variation in element size than the natural population (*P* < 0.001; R package *cvequality*, version 0.2.0, Marwick and Krishnamoorthy [2019]). Interestingly, the founding clone of this line shows four male peaks (fig. 4*d*), and indicated three elements of ∼34 Mb size. Thus, it appears that this same 34-Mb element causes all the observed genome size variation in the “large” selfed line, being present in different numbers in different offspring.

### Link between Independently Segregating Elements and Genome Size Variation

The hypothesis of independently segregating elements offers an explanation to many patterns in our data. However, does it provide a *sufficient* explanation for within-population genome size variation? To explore this idea, we formalized our findings in a quantitative model of multiple independently segregating elements (for details and code, see supplementary methods, Supplementary Material online). We assumed a basal diploid genome size at 414 Mb and that each additional element in a clone would proportionally increase its genome size. Thus, the basic input parameters and variables of the model were: 1) the basal genome size (i.e., 414), and 2) a vector describing the size and number of individual elements in a clone (e.g., [34 34 20] for two 34 Mb and one 20 Mb element, respectively). We assumed that all elements segregate completely independently from each other. Thus, if a clone contains, for example, five elements, there is a small chance that it produces males with zero or five elements, while the majority of males will contain two or three elements. In the model, we also defined a parameter for the precision of the flow cytometry measurement, in terms of the CV. We set its default value to 2.7%, reflecting the overall mean in our male peak data (supplementary table 3, Supplementary Material online), but we also explored a CV of 2%, which we obtained in some of our best samples. On the whole, our model can reproduce virtually all male peak patterns found in *B. asplanchnoidis*, and it illustrates some technical issues, for example, how measurement precision limits the detection of the fine patterns produced by small elements or when a mixture of different element sizes are present in a clone (supplementary figs. 7 and 8, Supplementary Material online). We could also reproduce the peculiar case of clone OHJ72, our outlier of the natural OHJ-population, with its extremely broad male peak. According to our model, this clone might harbor eleven 34-Mb elements, or even more, if these elements are smaller.

**Fig. 6.**
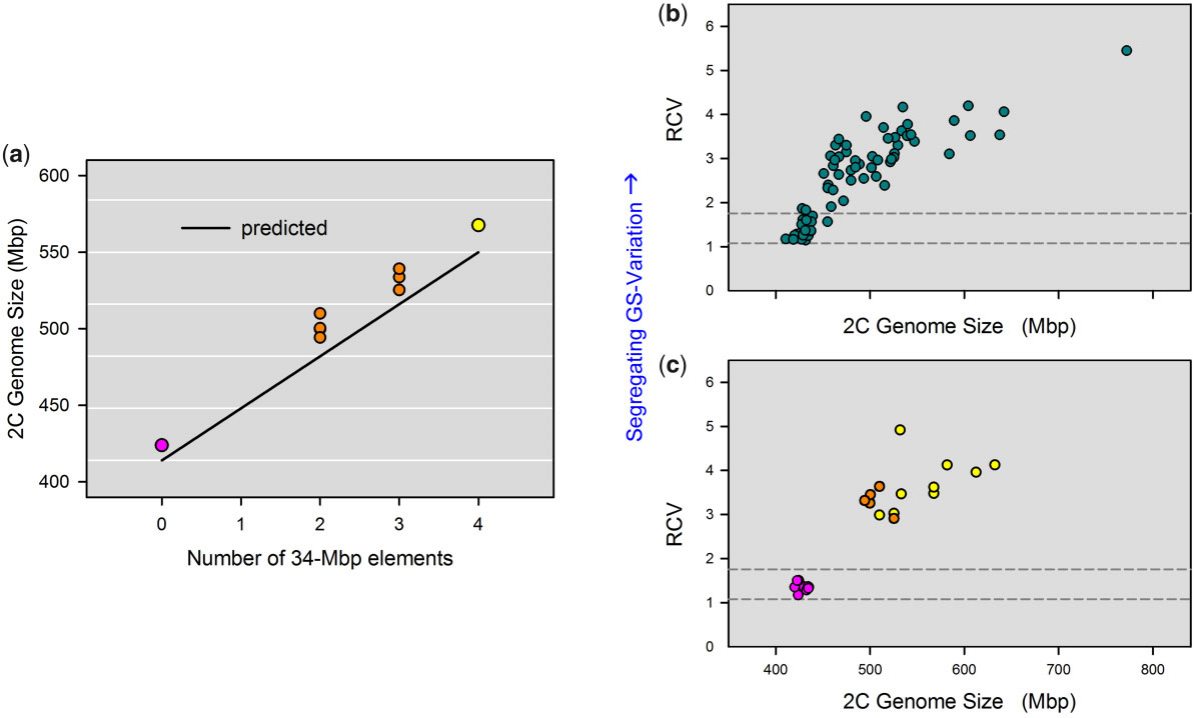
—Mechanistic explanations of intraspecific genome size variation. (*a*) Prediction of genome size based on the size and number of independently segregating elements, assuming a basal genome size of 414 Mb (i.e., free of elements). This figure shows seven clones of the inbred line cross (orange), from which we could determine the exact number of 34 Mb elements based on their MP patterns. The two parental clones, one with four elements (yellow) and the other with zero elements (purple), are also shown. (*b*) Genome size versus “relative coefficient of variation” (RCV) in clones of the natural population (fig. 1*a*) and their outbred descendants. We used RCV as a proxy for the amount of genomic material that independently segregates during meiosis. RCV was calculated by dividing the CV of all combined male genome sizes (in a clone) by the CV of the female genome size. Horizontal dashed lines are the 95% confidence intervals of the RCV in four outgroup species, which have no intraspecific genome size variation (*Brachionus rotundiformis*, *B. plicatilis*, *B. manjavacas*, B. “Nevada”). (*c*) Genome size versus RCV in selfed lines. Selfed line with small genome size in purple, line with large genome size in yellow, and the cross between the two inbred lines in orange.

One method to test whether independently segregating elements provide a sufficient explanation to genome size variation is to “predict” genome size of a clone based on the number and size of its elements. Although this prediction is recursive, since it requires knowledge of female genome size for sizing of the elements, it is not circular, because the distance between male peaks is free to vary. Thus, if we should find that predicted and observed genome sizes differ greatly, this would suggest that independently segregating elements are *not* a sufficient explanation for genome size variation. To explore this idea, we analyzed the crossed offspring of the two selfed lines (cf., fig. 3). This cross was especially suitable because of their low diversity of segregating elements (fig. 5*b* and supplementary table 2, Supplementary Material online). We obtained striking agreement between predicted and observed genome sizes, and could account for 96% of the diploid genome size, on an average, just based on the number of 34 Mb elements (fig. 6*a*). Nevertheless, all observed genome sizes were ∼18 Mb higher than the predicted ones, indicating that there might still be some variation that the model does not account for.

Identifying the exact size and number of independently segregating elements is not possible for the natural OHJ population as a whole, due to the higher diversity of segregating elements in this population (fig. 5*b*), and due to limited resolution of male peak patterns in clones containing multiple differentially sized elements (supplementary fig. 7, Supplementary Material online). To circumvent these limitations, we tested whether the amount of meiotically segregating genome size variation is correlated with genome size. To this end, we defined the variable RCV, which is the CV of all combined male genome sizes of a clone divided by the CV of the female genome size. Thus, RCV accounts for differences in measurement precision across samples (indicated by the CV of the female peak), while it requires only that a clone can produce males in order to be measured, thus extending our analyses to many more clones (*n* = 72). We found that the RCVs of OHJ-clones were considerably higher than those of outgroup rotifer species without genome size variation (fig. 6*b*), and that genome size and RCV were positively correlated (Spearman rank correlation, ρ  =  0.89, *P *<* *0.001). Consistently, RCVs in the “large” selfed line were significantly higher than in the “small” selfed line (fig. 6*c*; Mann–Whitney *U* test, *W* = 88, *P *<* *0.001). Our model predicted the same curvilinear relationship between genome size and RCV as we observed in *B. asplanchnoidis*, assuming either 20 or 34 Mb elements (supplementary fig. 9, Supplementary Material online). In particular, the prediction based on 20 Mb elements seemed to match the majority of clones of the OHJ population. Altogether, the positive correlation of RCV with genome size as well as the congruence of the model predictions and the natural OHJ-population suggests that independently segregating elements are largely responsible for genome size variation in *B. asplanchnoidis*.

## Discussion

### Extensive within-Population Genome Size Variation in *Brachionus asplanchnoidis*

Here, we report a case of within-species genome size variation in the rotifer *B. asplanchnoidis* and show that even individuals *within* a natural population can differ by 1.33-fold, exhibiting genome sizes ranging from 414 to 552 Mb. Moreover, artificial selection for large genome size can extend this range to 1.55-fold (640 Mb) within just two generations. To our knowledge, these ranges represent some of the most pronounced cases of intraspecific variation in animals and plants (supplementary table 4, Supplementary Material online).

Claims of intraspecific genome size variation have sometimes met skepticism, due to possible methodological artifacts (Greilhuber 2005), or the species concept underlying the study (Murray 2005). Thus, we used two different internal standards in our flow cytometry measurements, either *Drosophila melanogaster* flies or other rotifer clones that were coprepared with a focal sample, to demonstrate genome size differences among rotifer clones. Both internal standards yield virtually identical results, confirming that our genome size estimates are accurate and reflect real differences among clones (supplementary figs. 10 and 11, Supplementary Material online). Previous studies have documented the species status of *B. asplanchnoidis* (Michaloudi et al. 2017; Riss et al. 2017), and have shown that the populations from Austria, Mongolia, and Lake Nakuru are genetically distinct, but also experience natural gene flow on a larger geographic scale (Riss et al. 2017). Thus, in the case of *B. asplanchnoidis*, we can exclude any unrecognized cryptic species.

### Independently Segregating Elements Are Responsible for Genome Size Variation

Within-population variation of genome size in *B. asplanchnoidis* is mediated by multiple, large genomic elements that segregate independently during meiosis. These elements range between 15 and 34 Mb in size and are present in variable numbers in individuals of the natural population. Thus, they account for the full range of genome size variation in this population, including the apparent “outlier” at 792 Mb. Individuals with the smallest observed genome size are free of such elements, while individuals with larger genomes contain progressively more elements, in different numbers and combinations. Altogether, this allows for a seemingly gradual distribution of genome sizes across the whole population.

The meiotic behavior of these elements is unusual in that they segregate independently from each other. This has important implications for maintenance of genome size variation in this population, because it facilitates the (re-)generation of a large number of genome size variants in sexual offspring, even if the two parents have identical genome size. For example, consider two parents with three 20 Mb and two 30 Mb elements, respectively. Both have the same genome size (414 + 60 = 474 Mb), but due to independent meiotic segregation of these elements, and recombination, their crossed offspring can have one of ten different genome sizes and range between 414 and 534 Mb. There is some resemblance to allelic recombination in a genetically controlled trait determined by multiple loci. However, in the case of genome size, variation can even be generated in genetically highly homogeneous lines, such as selfed lines.

On a chromosome level, independently segregating elements in *B. asplanchnoidis* may resemble supernumerary chromosomes (B-chromosomes) that do not pair during meiosis. This is suggested by our results from the selfed line, which seems to have accumulated up to six identical elements derived from an ancestor with three elements. If so, the B-chromosomes are quite large in their relative size, since one 34 Mb element corresponds to 8.2% of the basal diploid base genome observed in this population. B-chromosomes reported in previous studies were often smaller than normal chromosomes (Jones et al. 2008), but there are also reports where they reach 3–5% of the diploid genome size (Jones et al. 2008; Ruiz-Ruano et al. 2011). On the other hand, regarding absolute size, B-chromosomes of other species can be twice as large the whole genome size of *B. asplanchnoidis* (Ruiz-Ruano et al. 2011). A second possible mechanism is that normal chromosomes carry large heterozygous insertions, that is, one chromosome carries an insertion while the other chromosome does not. According to this hypothesis, simple patterns of two or three male peaks could be caused by one or two elements located on different chromosome pairs, being present only once per chromosome pair.

Currently, there is no karyological information available that would allow us to distinguish between B-chromosomes versus heterozygous insertions into A-chromosomes as the cytological mechanism behind independently segregating elements. Only one study so far reported chromosome numbers for the genus *Brachionus*, and found 2*n* = 22 and 2*n* = 25 for two unidentified species of the *B.plicatilis* species complex (Rumengan et al. 1991). We have therefore endeavored to adopt several karyotyping protocols established for other invertebrates (Zadesenets et al. 2016; Guo et al. 2018), which involved squash preparations or cell suspension techniques in combination with DAPI staining of nucleic DNA. Unfortunately and despite large efforts, we did not obtain enough metaphase plates in *B. asplanchnoidis* for unambiguously assigning chromosome numbers to individual rotifer clones, let alone for quantifying subtle karyological differences among clones. This was likely caused by a lack of mitotically active tissues, since all cell divisions in these eutelic organisms are limited to a short period when the embryo is encapsulated in a robust eggshell (Nogrady et al. 1993).

Even though we cannot completely rule out that independently segregating elements could be integrated in the regular set of chromosomes, supernumerary chromosomes appear to be the most plausible explanation for our selfed lines. Since selfing leads to a 50% loss of heterozygous sites per generation (Falconer and Mackay 1996), heterozygous insertions into normal chromosomes should be either fixed or lost, and both outcomes should lead to a rapid decrease of segregational variation in subsequent generations of selfing. In contrast, we found that segregational variation for genome size increased and remained high in subsequent generations of selfing. Nevertheless, the two mechanisms are not mutually exclusive. For example, in maize, B-chromosomes and “heterochromatic knobs” on normal chromosomes can both contribute to intraspecific genome size variation (Smarda and Bures 2010).

Regardless of whether these independently segregating elements are located on supernumerary chromosomes or integrated in the regular set of chromosomes, they are clearly “dispensable,” since several clones of the OHJ-population did not possess any independently segregating elements at all. Thus, such elements probably do not contain important genes or regulatory sequences. Furthermore, since the genome of *B. asplanchnoidis* consists of at least 44% repetitive DNA sequences, in particular transposons (Blommaert et al. 2019), it seems plausible that the latter are a major constituent of such elements. Our present and ongoing research addresses this hypothesis via comparative genome sequencing of multiple clones of the OHJ-population.

### Toward a General Model of within-Species Genome Size Variation

The model developed here requires as parameters only a basal genome size (for individuals free of elements), a vector describing size, and number of all elements within a clone, and information about their meiotic segregation patterns. This model is consistent with high heritability of genome size and the strong response to selection, while it accounts for the fact that we could not select below a basal genome size. It is worth mentioning that the basal genome size that we defined for our population was not obvious from the distribution of the natural population (fig. 1*a*), but could only be established by directional selection (fig. 2). Interestingly, previous reports of intraspecific genome size variation in other organisms also report positively skewed distributions (Šmarda et al. 2008; Huang et al. 2014), which could be an indication of a basal genome size. Our model could reproduce virtually all observed meiotic segregation patterns (“male peaks”), and it is consistent with the increase of male segregational variation (RCV) with genome size that we observe in the OHJ population (fig. 6*b*). Finally, this model can explain the unexpectedly high genome size variation in a selfed line derived from a clone with large genome size.

Our model differs in notable aspects from models on genome size that were adopted from a perspective of QT models. Importantly, QT models are defined in terms of the mean genome size of a population governed by a large number of insertion and deletion alleles (Bilinski et al. 2018). In contrast, the point of reference in our model is the basal genome size, that is, the smallest attainable genome size given the standing genomic variation of a population/species. In some cases, this is equivalent to the genome size of the individual with the smallest genome, but there are exceptions. For example, it is possible that even individuals with smallest genomes in a population contain extra genomic elements that would segregate during meiosis, and crossing such individuals would result in at least some offspring with even smaller genomes. QT models thus refer to the special case where all members of a population contain some extra genomic elements and are above the basal genome size. It is important to realize that the basal genome here is not equivalent to a biologically minimum genome, for example, in a (hypothetical) specimen that is free of any noncoding DNA. Rather, the basal genome includes all DNA-additions that are already “fixed” on a population level, and thus do not contribute to within-species genome size variation any more. An interesting corollary is that any new deletion mutation will not only introduce a new variant to a population but also slightly decrease the value for the basal genome size.

Our present model can generalized even further. Currently, it is strongly oriented toward a mechanism that involves completely independent segregation of extra DNA, which might be best represented in the case of B-chromosomes. However, it can be extended to include other mechanisms, for example, where extra genomic material is located on normal chromosomes, either as a big contiguous insert, or as multiple smaller ones along a chromosome. Accounting for the latter would require some constraints regarding segregation patterns of extra DNA as such insertions will tend to cosegregate and thus appear as one large “element,” whose size corresponds to the net-difference of cumulative insert length between the two chromosomes. Such a generalization has interesting consequences. For example, extra genomic material may become fixed in a population once all members carry the same DNA inserts on both chromosomes, and accordingly, the basal genome size of that population would shift to a higher level. Ultimately, a general model would incorporate both mechanisms, integration to normal chromosomes and supernumerary chromosomes, and perhaps even an exchange between both pools (Araujo et al. 2001; Jones and Houben 2003; Houben et al. 2014; Navarro-Domínguez et al. 2017). Thus, genome size could be highly dynamic on short time scales, due to mechanisms that involve independent segregation, while fixation/loss of extra genomic material on the normal set of chromosomes could explain the long-term changes in genome size across populations or species. Overall, this model does not even contradict long-term gradual (Brownian motion) changes in genome size, while it adds a population-level perspective to taxa that seem to have undergone “saltations” in genome size over macroevolutionary time scales (Liedtke et al. 2018).

## Conclusions

We have shown that within-population genome size variation in the rotifer *B. asplanchnoidis* is mediated by relatively large genomic elements that segregate independently of each other during meiosis. Our data on short-term artificial selection and inbred line variation suggest that a model that involves only two variables, a basal genome size and a vector specifying the number, size, and segregation behavior of the elements that increase genome size is sufficient for capturing most aspects of genome size dynamics in this population. Collectively, our study closes an important gap in our knowledge of how intraspecific genome size variation in populations is mediated by processes at the individual level. Since the genome size variations in this model system are realized across a relatively homogeneous genomic background, this has general implications for identifying the evolutionary forces that are responsible for the immense variation of genome sizes seen across eukaryotes. Most notably, future studies in this system should allow disentangling whether larger genome size can be beneficial, or whether it is always slightly deleterious. In the future, it will also be interesting to elucidate in more detail the mechanisms behind these independently segregating elements, for example, their underlying genomic architecture, or how they interact with the “nondispensable” parts of the genome. 

## Supplementary Material

Supplementary data are available at *Genome Biology and Evolution* online.

## Supplementary Material

evz253_Supplementary_Data
