# Supplementary material for: Within-Population Genome Size Variation is Mediated by Multiple Genomic Elements That Segregate Independently during Meiosis
Source: Genome Biol Evol. 2019 Nov 19;11(12):3424–35. doi: 10.1093/gbe/evz253 (PMC7145553; doi:10.1093/gbe/evz253)
Supplement: evz253_Supplementary_Data [file evz253_supplementary_data.zip › SupplementaryInformation.docx]

# Summary of all supplementary information

**Supplementary information**

Supplementary Figures 1–11, Supplementary Table 4, and Supplementary Methods.

**Supplementary file**

Supplementary table 1: Genome size estimates of all clones used in this study; 2: Clones used for sizing of independently segregating elements; 3: Clones used for RCV (relative coefficient of variance) calculations.

# Supplementary Information

## Supplementary Figures

**
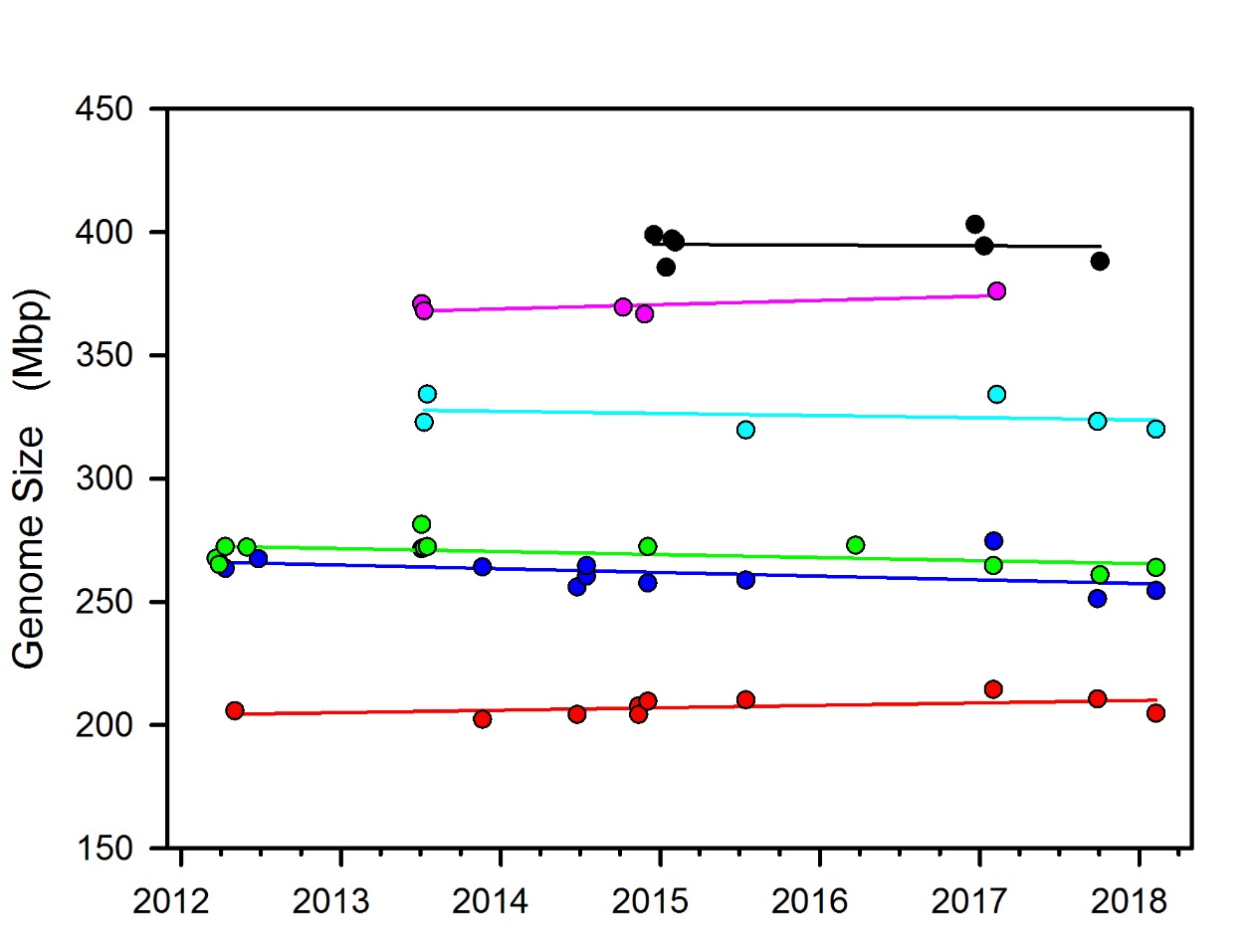
**

**Supplementary figure 1. Long-term constancy of genome size during clonal propagation.** Time courses of genome size for six *B. asplanchnoidis* clones over a period of > five years (i.e., approximately 600 asexual generations). Colours denote different clones: *ohj22* (red), *ohj7* (blue), *ohj13* (green), *mnchu24* (cyan), *mnchu008* (pink), *ohj72* (black). Each symbol represents an independent genome size measurement.


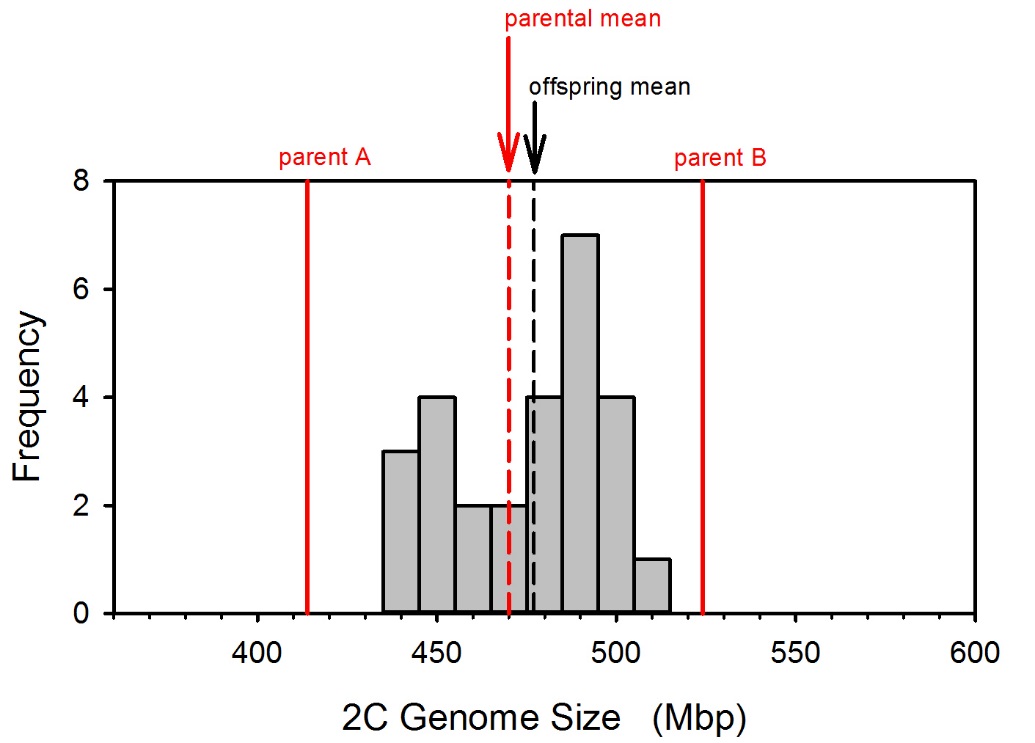


**Supplementary figure 2.** **Sexual cross between a clone with large genome size and a clone with small genome size.** Parental genome sizes (414 and 524 Mbp) are indicated by the vertical red lines. The dashed red line indicates the parental mean, while the dashed black line indicates the offspring mean genome size. Overall, the 27 offspring genome sizes were intermediate between their parents, even though their distribution was not unimodal.


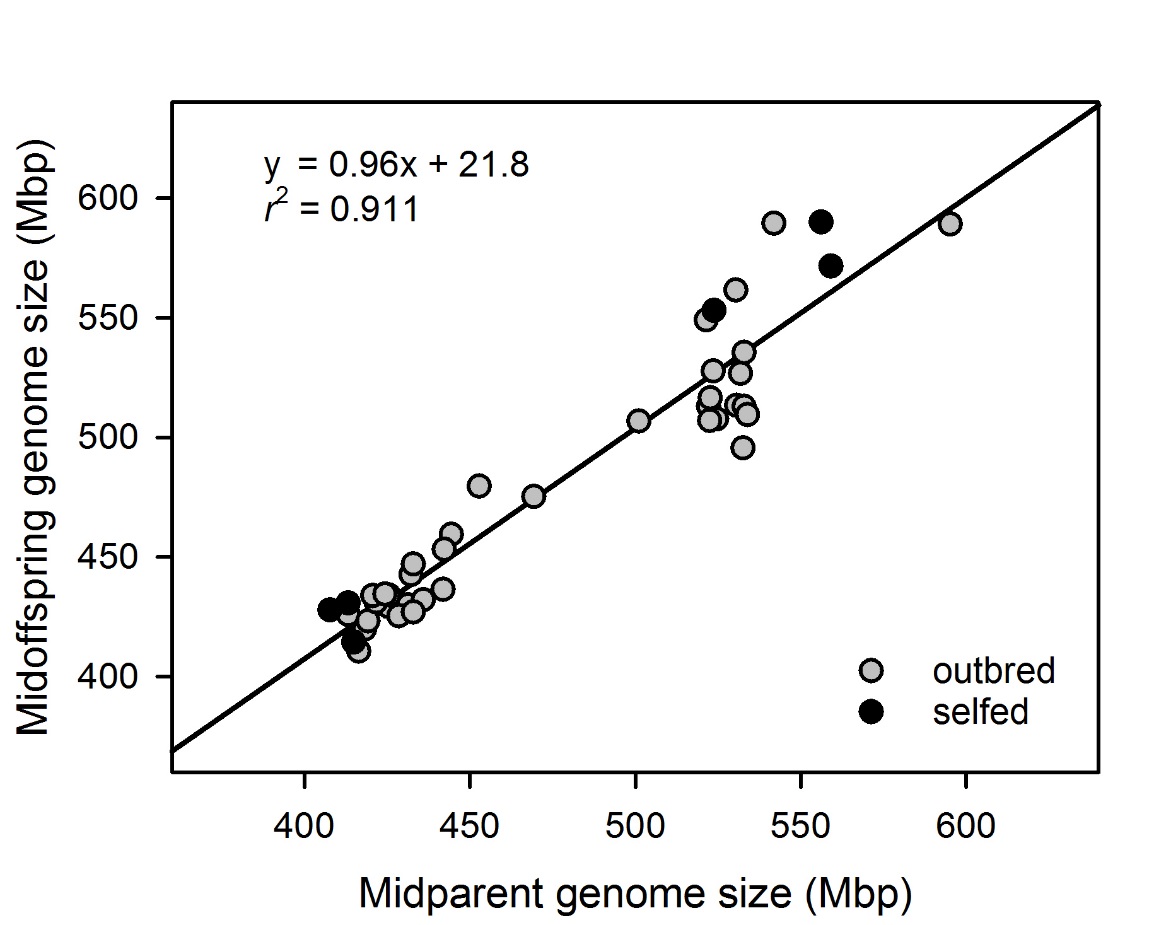


**Supplementary figure 3.** **Narrow-sense heritability (*h*^2^) of genome size in *B. asplanchnoidis*.** Each symbol represents the mean of parents and offspring genome sizes of a family. This figure combines our results from the artificial selection experiment and crossings between clones with large and small genome size.


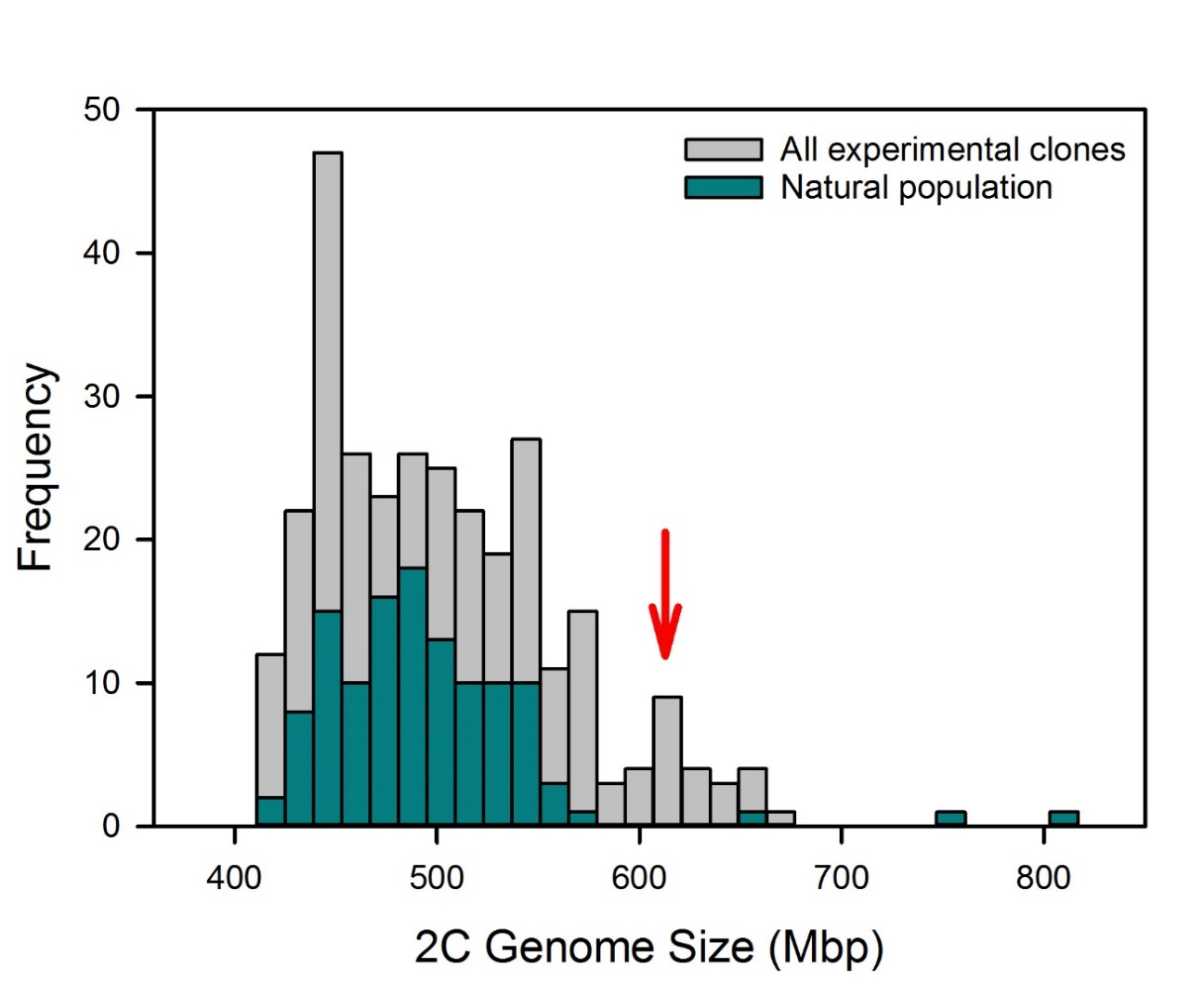


**Supplementary figure 4. Distribution of genome sizes found in *B. asplanchnoidis*.** **Gray**: All *B. asplanchnoidis* clones measured so far (including natural populations, selection lines, and selfed lines). **Green**: Only the natural OHJ population. **Red arrow** highlights genome size classes absent in the natural population, which could be artificially selected in the laboratory.


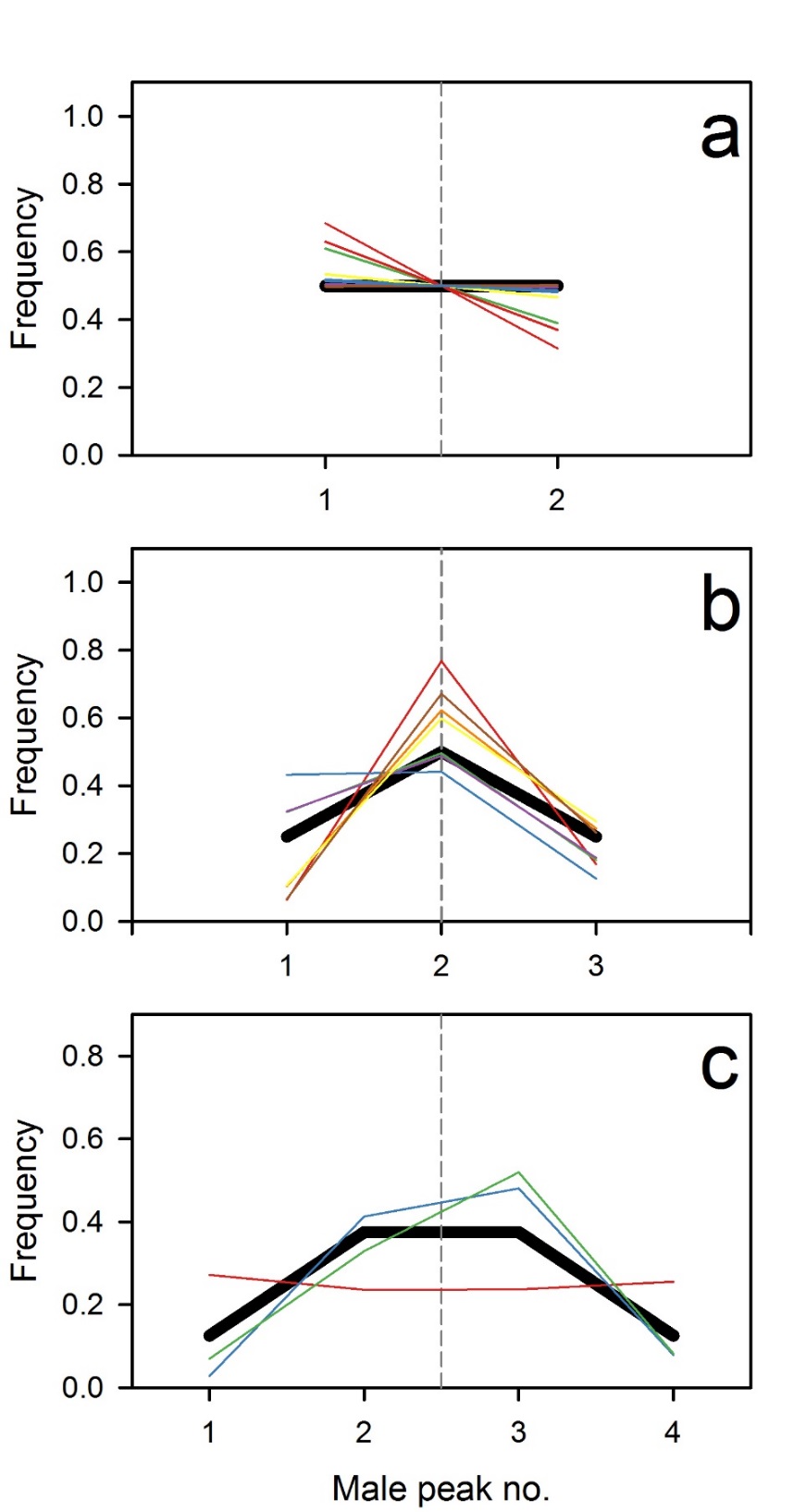


**Supplementary figure 5.** **Relative frequency of the different male genome sizes.** **a** two malepeaks; prediction (thick line) based on one independently segregating element **b** three malepeaks; prediction based on two equally sized elements, **c** four male peaks; prediction based on three equally sized elements. Coloured lines denote different clones. Dashed vertical line indicates 0.5x diploid genome size.


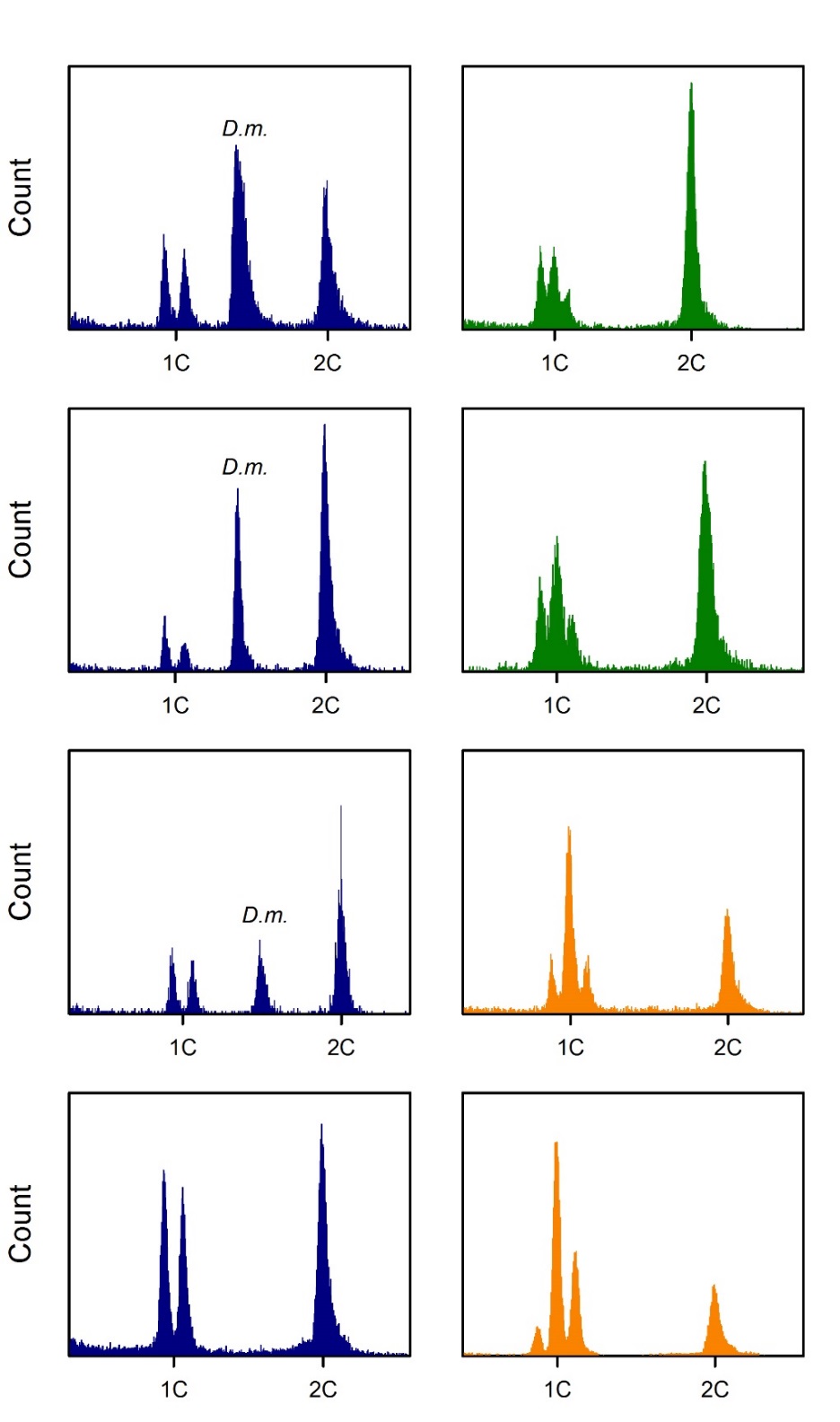


**Supplementary figure 6. Constancy of “male peak” (MP) patterns in asexually propagated clones.** The number of male peaks and their heights (relative to each other) are characteristic for a clone. This figure shows the 2C-female peaks and the corresponding male peaks (peaks scattering around 1C) for three different clones on different dates. **Blue**: Clone *k13x110n2* (two MPs); **Green**: Clone *ohj76* (three MPs); **Orange**: Clone *ohj85* (three MPs). In some of these measurements we used *Drosophila melanogaster* (**D.m.**) as internal standard. Please note that the height of MPs relative to the 2C-female peak might differ among dates, since the number of males per female in a culture is not always the same.


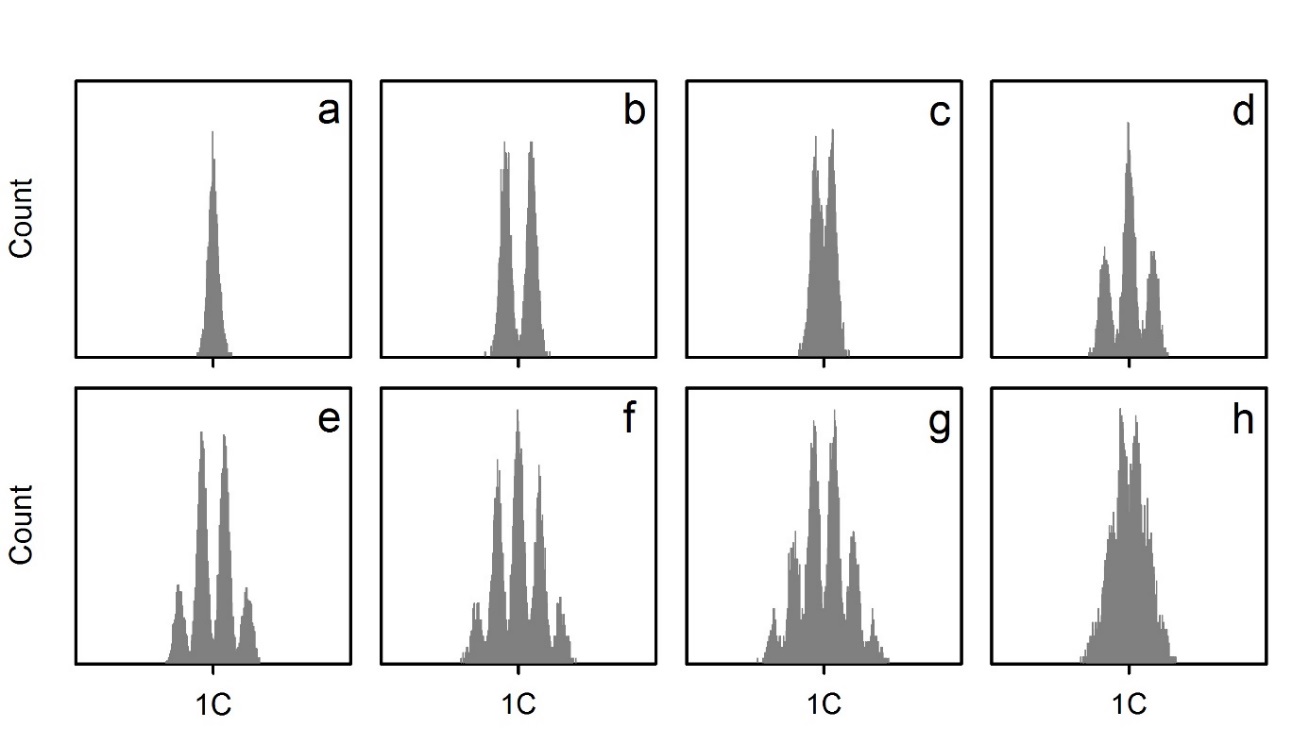


**Supplementary figure 7.** **Model simulations of “male peak” (MP) patterns with only one type of independently segregating element. a** No element; **b** one element of 34 Mbp size; **c** one element of 20 Mbp; **d** two elements of 34 Mbp; **e** three elements of 34 Mbp; **f** four elements of 34 Mbp; **g** five elements of 34 Mbp; **h** five elements of 20 Mbp. The model-parameter ‘measurement precision’ was set to 2.7% (i.e., the coefficient of variance) in all cases.


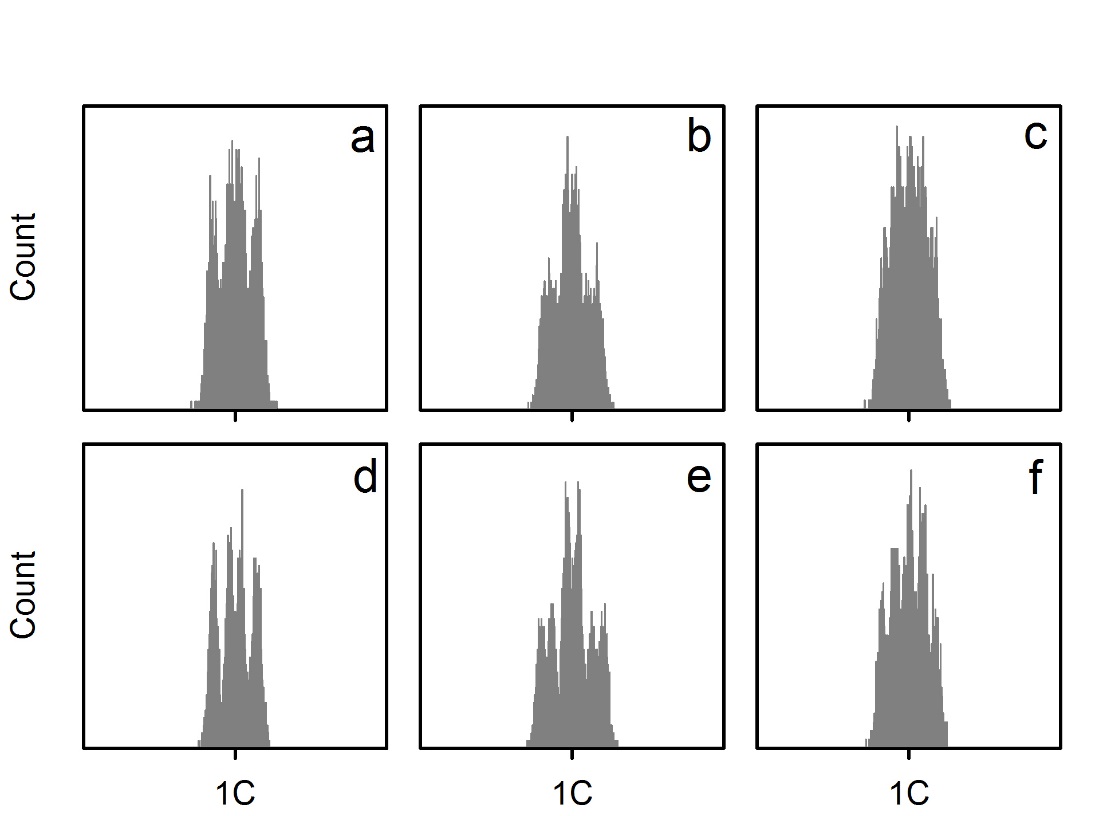


**Supplementary figure 8.** **Model simulations of “male peak” (MP) patterns with different types of independently segregating elements.** **a** two elements with 20 and 34Mbp; **b** three elements with 15, 34, and 34 Mbp; **c** three elements with 15, 20, and 34 Mbp. The model-parameter measurement precision was set to 2.7% (i.e., the coefficient of variance). **d-f** are identical to **a-c**, except that measurement precision was set to 2.0%.


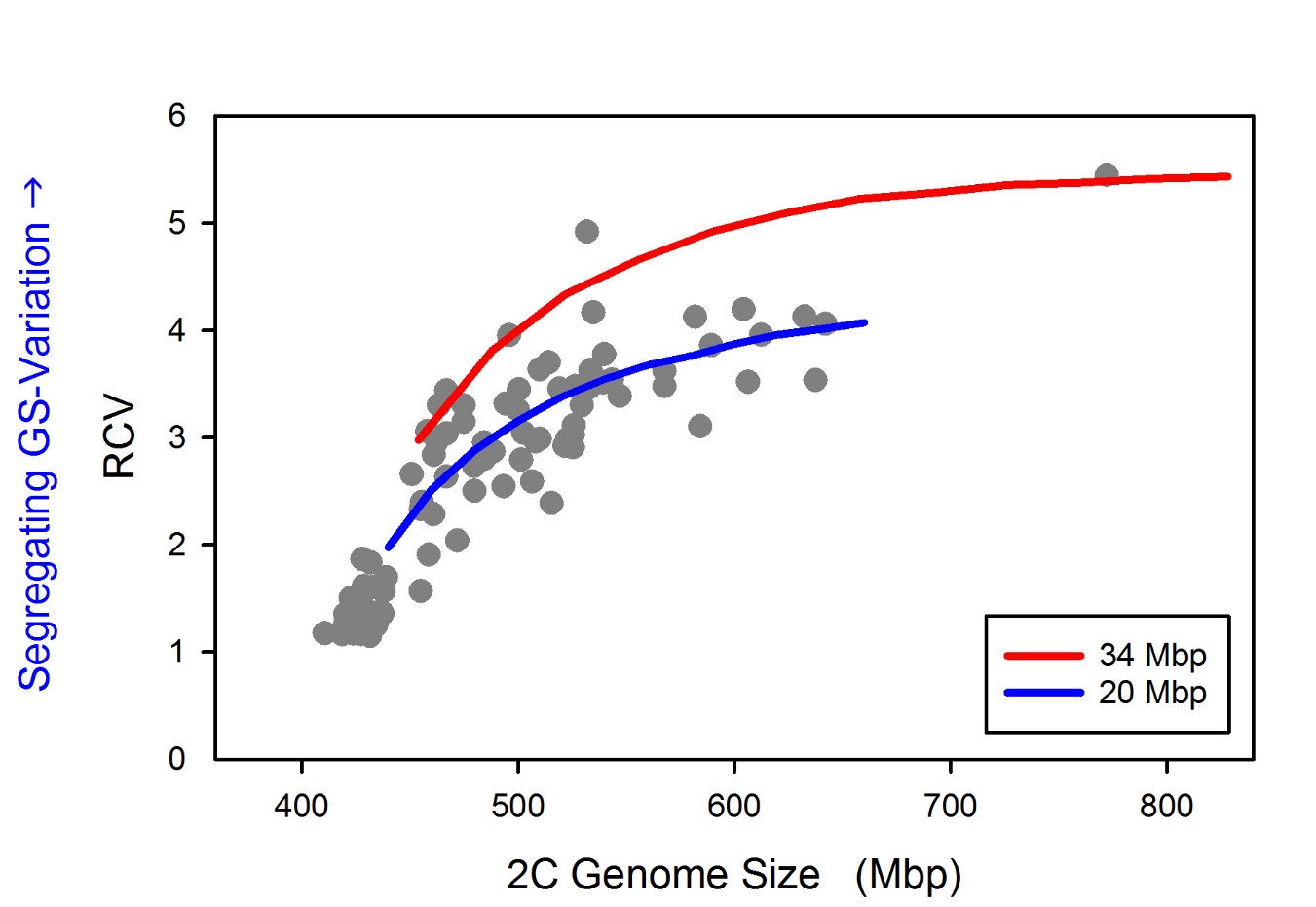


**Supplementary figure 9.** **Model simulations of the relationship of RCV and genome size**. RCV (“relative coefficient of variation”) was calculated by dividing the CV of all combined male genome sizes (in a clone) by the CV of the female genome size. Red line shows model predictions for genomes with multiple 34 Mbp elements. Blue line shows model predictions for genomes with multiple 20 Mbp elements. . The model-parameter ‘measurement precision’ was set to 2.7%. Dots are all measurements of RCV in this study (cf. Fig. 6 b & c)


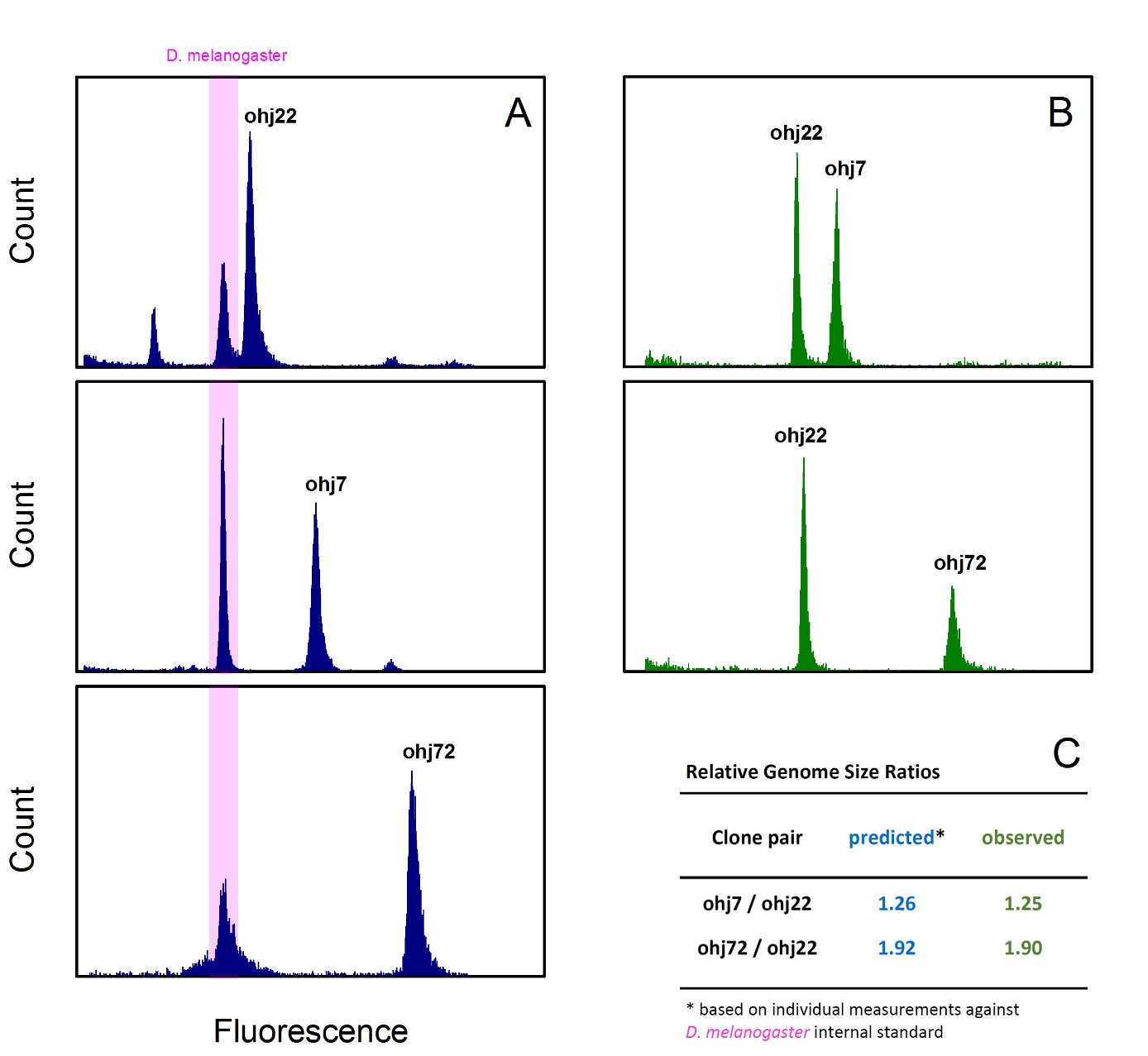


**Supplementary figure 10.** **Comparison of two variants of the flow cytometry method for measuring intraspecific genome size variation.** **a** Three clones of different genome size were co-prepared with an internal standard of known genome size (*D. melanogaster*, strain ISO 1, 352 Mbp). **b** Pairs of the same three clones were combined into a sample. **C** A comparison of the genome size ratios (= large GS / small GS) determined by the second method shows that these closely matched those predicted by the measurements of the individual rotifer clones, as determined by the first method.

**
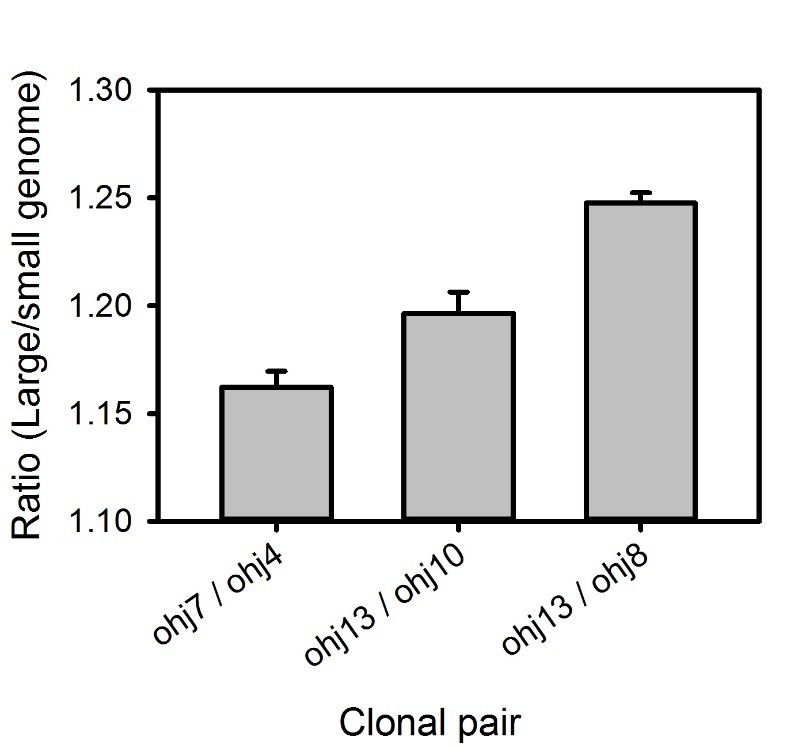
**

**Supplementary figure 11.** **Genome size differences among selected pairs of clones in the OHJ-population.** Error bars are standard deviations based on 3-4 replicates (measured on different dates). In each replicate the clonal pairs were co-prepared and stained in the same tube. Kruskal-Wallis test: *H*= 8.95, *d.f*.=2, P=0.011.

## Supplementary Tables

**Supplementary table 4.** Examples of natural intraspecific genome size variation in animals and plants

|  |  |  |  |  |
| --- | --- | --- | --- | --- |
| **Species name** | **Common name** | **1C-value (pg)** | **Fold-variation in GS** | **Reference** |
|  |  |  |  |  |
|  |  |  |  |  |
| *Zea mays* | Maize | 2.30 | 1.37 | Diez et al. (2013) |
| *Festuca pallens* | Grass | 2.53 | 1.19 | Smarda et al. (2008) |
| *Microseris douglasii* | Coastal silverpuff | 1.20 | 1.20 | Smarda & Bures (2010) |
| *Hordeum spontaneum* | Wild barley | 5.50 | 1.13 | Smarda & Bures (2010) |
| *Arabidopsis thaliana* | Thale cress | 0.16 | 1.14 | Long et al. (2013) |
|  |  |  |  |  |
| *Eyprepocnemis plorans* | Grasshopper | 10.39 | 1.19* | Ruiz-Ruano et al. (2011) |
| *Synalpheus duffyi* | Snapping shrimp | 17.38 | 1.35 | Jeffery et al. (2016) |
| *Drosophila melanogaster* | Fruit fly | 0.18 | 1.15† | Huang et al. (2014) |
| *Brachionus asplanchnoidis* | Rotifer | 0.21 | 1.33 (1.91) | **this study** |
|  |  |  |  |  |
| * assuming individuals with zero *vs*. six B-chromosomes of 0.64pg size | | | |  |
| † variation between inbred strains | |  |  |  |
|  |  |  |  |  |

## Supplementary Methods

### Rotifer cultures

Rotifers were cultured in F/2 medium {Guillard, 1962 #14} at 16 ppt salinity and with *Tetraselmis suecica* algae as food source (500–1000 cells μl^−1^). Continuous illumination was provided with daylight LED lamps (SunStrip, Econlux) at 30–40 µmol quanta m^−2^ s^−1^ for rotifers, and 200 µmol quanta m^−2^ s^−1^ for algae. During the experiments, rotifers were cultured at 23°C. Stock cultures were kept either at 18 °C, re-inoculated once per week by transferring 20 asexual females to 20ml fresh culture medium, or they were kept for long-term storage at 9°C, replacing approximately 80% of the medium with fresh food suspension every 4 weeks.

### Genome size measurements

To determine the genome size of rotifer clones, we used flow cytometry and propidium iodide staining of nuclei. Rotifer clones were grown in 250-1000 ml flasks until their population size reached several hundreds to a few thousand individuals. On the day before preparation, rotifers were harvested with 60 µm sieves, and starved overnight in 11 ppt 0.2µm-filtered medium, followed by two washes with filtered medium on the next day. For each sample, we isolated 350 females and subjected them to the flow cytometry protocol. Briefly, rotifers were homogenized in a citrate buffer (3.4 mM Trisodium citrate dihydrate, Nonidet P40 at 0.1% v/v, 1.5 mM Sperminetetrahydrochloride, 0.5 mM Trishydroxymethylaminomethane, pH 7.6), and were then transferred to 750 μl stock solution in a 1 ml Dounce tissue homogenizer. Rotifers were homogenized on ice with 20 strokes using the “tight” pestle of the homogenizer. As internal standard of known genome size we used the fruit fly, *Drosophila melanogaster* (strain ISO-1, nuclear DNA content: 0.35 pg, {Gregory, 2019 #459}). After adding two female *Drosophila* heads, the sample was further homogenized with ten strokes. Large debris was removed by filtration through a 40 μm mesh nylon sieve. After addition of 100 μl of 0.021% Trypsin (dissolved in stock solution) the sample was incubated for exactly 10 min at 37°C. To prevent further degradation, 75µl of 0.25% trypsin inhibitor was added (this solution also included 0.05% RNAse A) and the samples were incubated for another 10 min at 37°C. Finally, samples were stained with propidium iodide at a concentration of 50 μg/ml. Stained samples were kept overnight on ice in the dark. Flow cytometric analysis was performed on the next day on an Attune NxT^®^ acoustic focusing cytometer (Thermo Fisher) with an excitation wavelength of 561 nm and a custom-made 590–650 nm bandpass filter (yellow, YL-2) for detection of propidium iodide fluorescence. Flow cytometric data were analyzed using FlowJo software version 10.0.7r2 (FlowJo LLC). To exclude doublets (i.e., nuclei that pass the detector too close together, thus being recorded as a single “event”) we employed YL2-A *vs*. YL2-H gating. Coefficients of variance (CVs) of individual peaks typically ranged between 1.5% and 4% for both *Drosophila* and rotifers. Very few measurements had CVs higher than 5%, and those replicates were discarded. Conversion from picograms DNA to base pairs were made with the factor: 1 pg =978 Mbp {Gregory, 2019 #459}. In most cases, we obtained at least three replicate measurements for a clone, usually on different days and sometimes with several weeks or months apart.

### Crossings between rotifer clones

For sexual crosses between two rotifer clones, we used freshly hatched virgin females and males, which were harvested as eggs from dense rotifer cultures that had initiated sexual reproduction. Eggs were detached from the females by vigorously vortexing the rotifer culture in 50-ml Falcon tubes for ten minutes. Crossings between clones were accomplished by placing 100 female eggs and 50 male eggs together into the same well of a 24-well plate filled with 750 µl of F/2 medium. After 24 h, when all viable eggs had hatched and animals had time to mate, females were transferred to new wells with fresh food suspension. After a few days, when females started producing eggs, we classified them into asexual females, sexual male-producing females (which were unfertilized), and sexual resting egg producing females. All resting egg producing females were isolated and stored at 7 °C in the dark for at least 2 weeks. To induce hatching, resting eggs were incubated with food suspension at 23 °C and high light intensities (200 µmol quanta m^−2^ s^−1^). Usually after 48 h, the first hatchlings started to emerge, and clonal cultures were initiated.

### Theoretical model of independently segregating elements

We conceptualized a theoretical model to summarize our finding of independently segregating genomic elements. The main purpose of this model was to test the hypothesis that these genomic elements are *sufficient* as the mechanism of genome size variation. In particular, we examine whether such a model is consistent with our observations of genome size variations in males from clones of different diploid genome size. The basic input parameters and variables of the model are: (i) an assumed basal diploid genome size of 414 Mbp (for a clone that contains no additional genomic elements), and (ii) a vector describing the size and number of individual elements in a clone (e.g., [34 34 20] for two 34 Mbp and one 20 Mbp element, respectively). The output variable of the model was the predicted diploid genome size of females, calculated as basal genome size + the summed contribution of all elements. The second output variable was the genome size distribution of haploid males of this same clone (i.e., its “male-peak pattern”). Male genome sizes were calculated as ½ basal genome size+ the summed contribution of all elements in a particular male. To this end, we assumed that all elements segregate completely independently from each other during meiosis, i.e., each element having an equal chance of ending up in one out of four gametes after the two meiotic divisions. For instance, a clone with two 34 MBp elements should produce 25% gametes/males containing no element, 50% gametes containing one element (50%), and 25% containing two elements. We calculated the male genome size distributions for a total of 10,000 gametes/males per clone. We also simulated measurement errors by assuming a coefficient of variance of 2.7%. This value is based on the average precision of our flow-cytometry measurements of male genome size variation (Supplementary table 3). We also explored CV values of 2%, which we obtained in some of our best samples. The model was written in the MATLAB programming environment (MathWorks®, version R2017A) with the code as follows.

function [relCV, gsFem, allGametes, allGametesERR] = ...

estimateRelativeCV(Bvec, measErr)

% MATLAB FUNCTION "estimateRelativeCV" for calculation of (diploid) female

% genome size and male genome size distribution, based on a vector

% specifying the size and number of independently segregating elements in a

% clone.

%

% Claus-Peter Stelzer (2019)

% Input variables

% Bvec = [20]; % Vector of elements (contains size in MBp)

% measErr = 2.7; % measurement error in flow cytometry (coefficient of

% variance in %)

% Model parameters

baseGS = 207; % Genome size of male without elements

gsFem = baseGS*2 + sum(Bvec); % Genome size of diploid female

nGametes = 10000; % Number of gametes/males simutated

allGametes = nan(nGametes,1);

for i = 1 : 2 : nGametes-1

GameteA = baseGS;

GameteB = baseGS;

for j = 1:length(Bvec)

if rand>0.5

GameteA = GameteA + Bvec(j);

else

GameteB = GameteB + Bvec(j);

end

end

allGametes(i) = GameteA;

allGametes(i+1) = GameteB;

end

% Convert coefficient of variance into standard deviation

maleMeanGS = gsFem/2;

sdMales = measErr/100*maleMeanGS;

% Simulate with measurement error

allGametesERR = allGametes + sdMales*randn(size(allGametes));

mpCV = std(allGametesERR)/mean(allGametesERR)*100;

relCV = mpCV/measErr;
